# Ninjin’yoeito ameliorates deficits in self-care behaviors in a polyinosinic-polycytidylic acid-induced fatigue model via dopamine D2 receptor activation

**DOI:** 10.1007/s11418-026-02035-4

**Published:** 2026-06-02

**Authors:** Chihiro Yamada, Sachiko Mogami, Yoshiaki Sato, Hayato Baba, Tsutomu Fujii

**Affiliations:** 1 TSUMURA Kampo Research Laboratories, TSUMURA & CO., 3586 Yoshiwara, Ami-machi, Inashiki-gun, Ibaraki, 300-1192 Japan; 2 Department of Surgery and Science, Faculty of Medicine, Academic Assembly, University of Toyama, 2630 Sugitani, Toyama, 930-0194 Japan

**Keywords:** Poly(I:C), Fatigue, Ninjin’yoeito, Grooming, Dopamine

## Abstract

Fatigue is induced by physical and mental overload and decreases activity, motivation, and judgment, interfering with daily life. An association between fatigue and prolonged activation of the immune system has been suggested. However, the detailed underlying mechanisms are unknown, and effective treatments for fatigue have not been established. Ninjin’yoeito, a Kampo medicine, reportedly improves anorexia and fatigue scores. In this study, we examined the anti-fatigue effects of Ninjin’yoeito extract powder (NYT) in a polyinosinic-polycytidylic acid (poly[I:C])-induced mouse model of inflammatory fatigue. C57BL/6 male mice were intraperitoneally injected with poly(I:C) (12 mg/kg) to assess locomotive activity and nesting and grooming behaviors. NYT was orally administered to mice. NYT showed no effect on the reduction of locomotive and wheel-running activities following poly(I:C) administration. The enhanced rectal temperature and peripheral and hypothalamic levels of inflammatory cytokines also remained unaffected by NYT. In contrast, NYT significantly restored the nesting and grooming behaviors impaired by poly(I:C) administration. The restorative effect of NYT was abolished by cotreatment with a dopamine D2 receptor (D2R) antagonist. Furthermore, the impaired grooming behavior following poly(I:C) administration was restored by the dopamine-metabolizing enzyme monoamine oxidase (MAO)-B inhibitor, but not by the MAO-A inhibitor. Ingredients of NYT, such as formononetin derived from the astragalus root, paeonol from the peony root, heptamethoxyflavone and nobiletin from citrus unshiu peel, demonstrated inhibitory activities against MAO-B. These findings suggest that NYT improves fatigue-like self-care deficits potentially by inhibiting dopamine metabolism, likely via MAO-B, leading to enhanced D2R signaling.

**Graphical abstract:**

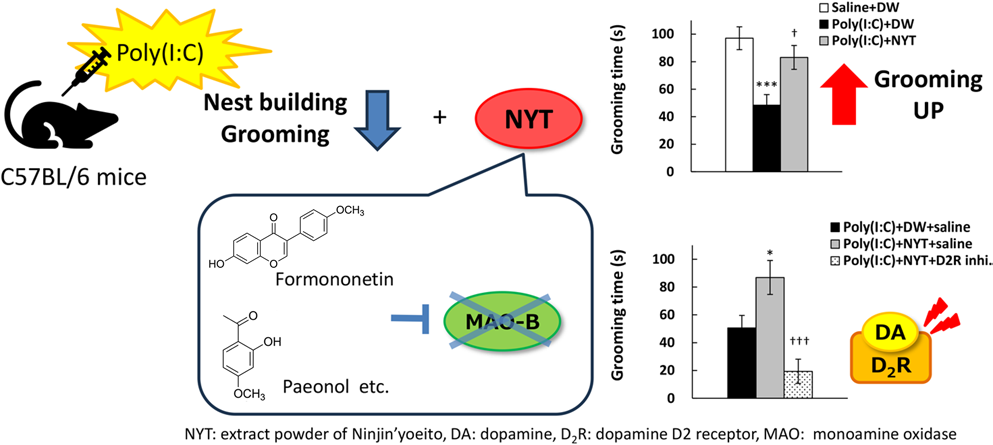

**Supplementary Information:**

The online version contains supplementary material available at 10.1007/s11418-026-02035-4.

## Introduction

Fatigue is indicated by feelings of weariness, tiredness, or lack of energy. It is attributed to physical and mental overload and reduces the capacity to perform daily activities [1–3]. High levels of fatigue severely affect the safety and health of workers, highlighting the need for preventing fatigue. Furthermore, fatigue can cause depression, anxiety, pain, cognitive decline, and decreased motivation [4–6]. Fatigue is also sometimes complicated by other diseases that may exacerbate fatigue symptoms and threaten the maintenance of physiological function.

While fatigue can be caused by various factors, prolonged activation of the immune system and systemic inflammation have been suggested as a cause of fatigue [7]. High concentrations of circulating pro-inflammatory cytokines have been reportedly associated with fatigue symptoms in patients with chronic fatigue syndrome [8–10], postoperative fatigue [11, 12], and cancer-related fatigue [13, 14]. Recently, fatigue has been reported as one of the most frequent (approximately 50%) typical clinical manifestations other than respiratory symptoms as a sequela following coronavirus disease-2019 [15, 16]. Viral infections cause acute inflammatory responses and the production of inflammatory cytokines [17]. These cytokines also affect the central nervous system, causing neuroinflammation, anorexia, increased sleepiness, decreased concentration, and other symptoms [16, 18], thereby affecting the whole body. Although rest is expected to temporarily mitigate fatigue, it is not a curative treatment, and no effective therapy has been established thus far.

Intraperitoneal administration of synthetic double-stranded RNA polyinosinic-polycytidylic acid (poly[I:C]) has been used to create models of fatigue induced by viral infection, chronic fatigue syndrome, or immunologically induced fatigue. The injection of poly(I:C) provokes immune-mediated inflammatory responses cascading into the production of inflammatory cytokines [7, 19, 20]. In rodents, poly(I:C) administration has been shown to increase body temperature and enhance depression-like behavior while reducing locomotive activity, wheel-running activity, body weight, and exploratory behavior [21–23]. Cytokines and neurotransmitter monoamines play important roles in these processes [23, 24].

Ninjin’yoeito, a Kampo medicine, is a drug approved under health insurance coverage by the Japanese Ministry of Health, Labour and Welfare for the management of decreased physical activity, fatigue, and loss of appetite. Ninjin’yoeito administration has been shown to improve the anorexia and fatigue visual analog scale scores in patients with mild cognitive impairment and Alzheimer’s disease [25]. Among severely ill COVID-19 patients admitted in an intensive care unit (ICU), the group that received Ninjin’yoeito administration had shorter ICU and hospital stays than the non-administered group [26]. We have previously reported that the extract powder of Ninjin’yoeito (NYT, 1.5 g/kg) ameliorates deficits in nesting behavior via dopamine D2 receptor (D2R) stimulation in mice exposed to water immersion stress [27]. Thus, NYT may affect dopamine receptors or dopamine levels and may improve psychiatric symptoms, fatigue, and decreased motivated activity; however, whether NYT has an anti-fatigue effect in an immunologically induced fatigue model is yet to be determined.

Therefore, in this study, we examined the effect of NYT in poly (I:C)-induced fatigue-like mouse model. Furthermore, we investigated the effects of the brain-penetrant ingredients of NYT on the enzymes that metabolize dopamine and other monoamines.

## Materials and methods

### Animals

Male C57BL/6J mice were purchased from The Jackson Laboratory Japan, Inc. (Yokohama, Japan) and were 8 weeks of age at the time of the experiments. The mice were housed in individual plastic cages in a room with controlled temperature (23 ± 3 ℃) and humidity (50 ± 20%) under a 12-h light (7:00 h–19:00 h)/12-h dark cycle, with free access to food and water. All the experiments were performed between 09:00 and 17:00. All animal care duties and experiments were performed in accordance with the Animal Care and Use guidelines issued by The Institutional Animal Care and Use Committee of TSUMURA & CO. (Tokyo, Ibaraki, Japan; approval nos. 18-077, 20-008, 21-015, 21-049, 22-010 and 24-005) and the ARRIVE guidelines.

### Test substances

**Fig. 1:**
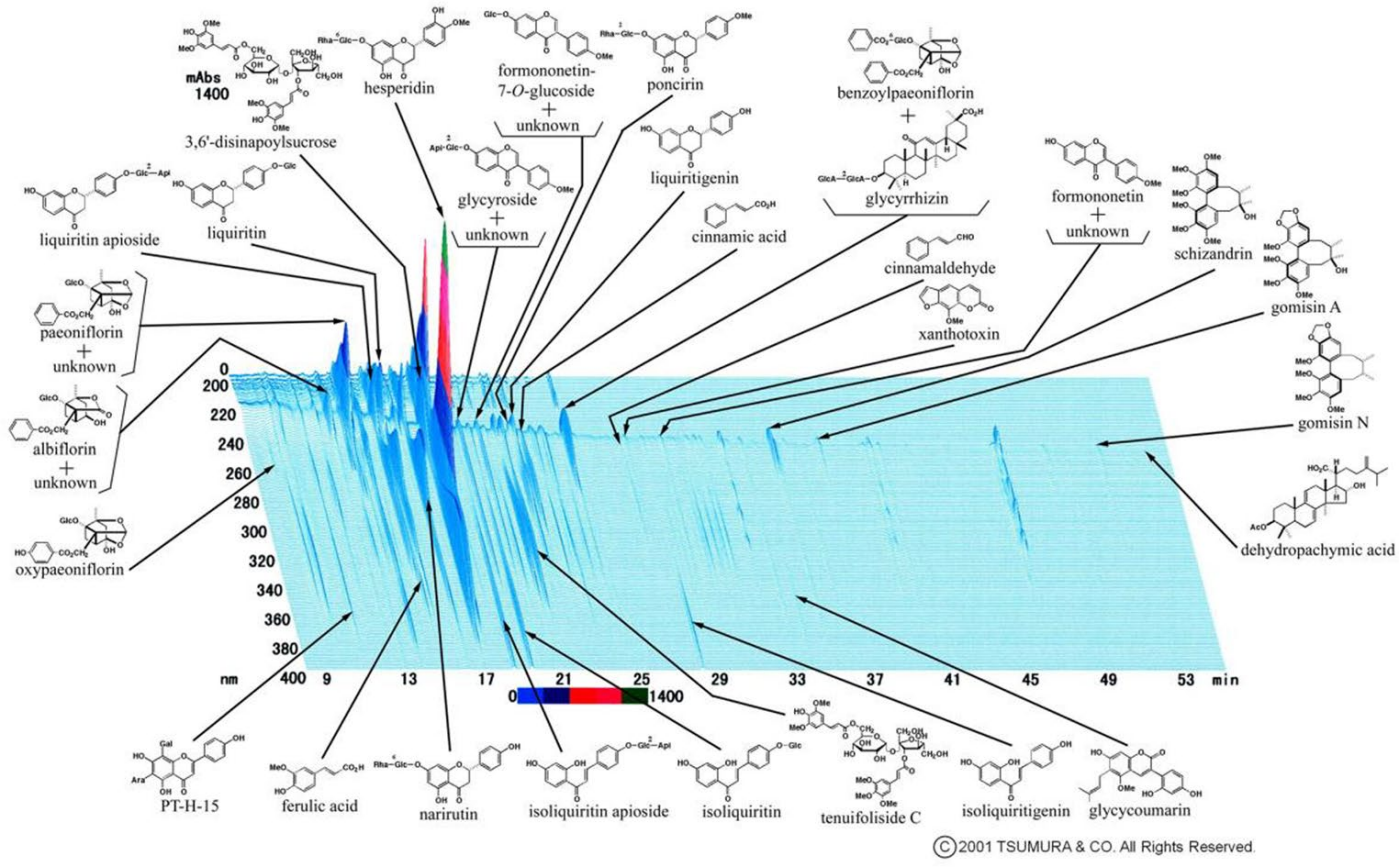
Three-dimensional high-performance liquid chromatography profile of Ninjin’yoeito (provided by TSUMURA & CO.)

High-molecular weight polyinosinic-polycytidylic acid (poly[I:C]) (InvivoGen, USA) was dissolved in saline according to the manufacturer’s instructions. NYT (extract powder) was manufactured by TSUMURA & CO. as an intermediate product (without excipients) of Ninjin’yoeito Extract Granules for Ethical Use, in compliance with good manufacturing practice requirements. Details of its preparation have been described previously [28]. Briefly, a mixture of the 12 crude drugs listed in Supplementary Table S1 was extracted with hot water and subsequently dried using a spray‑drying method. A three-dimensional high-performance liquid chromatography profile of NYT of a certain Lot provided by TSUMURA & CO. is shown in Fig. 1. NYT was suspended in distilled water and orally administered to mice at the dose of 1.5 g/kg, based on previously reported dose–response relationships [27, 29]. NYT was administered once daily from 3 days before until the day of poly(I:C) administration (Day 4, the day of assessment). The selective D2R antagonist metoclopramide hydrochloride (Sigma-Aldrich, USA), selective dopamine D1 receptor (D1R) antagonist SCH-23390 hydrochloride (Sigma-Aldrich), monoamine oxidase B (MAO-B) inhibitor pargyline hydrochloride (Sigma-Aldrich), and MAO-A inhibitor clorgiline (Sigma-Aldrich) were used as test drugs. Drugs were dissolved in sterile saline and administered at 10 ml/kg body weight. All drugs were administered at a dose that did not influence the body weight, food intake, and nesting or grooming behaviors in normal mice based on the results of a preliminary study and previous reports; metoclopramide 10 mg/kg [30, 31], SCH-23390 0.05 mg/kg [32, 33], pargyline 20 mg/kg [34], and clorgiline 5.0 mg/kg [35].

### Evaluation of locomotive activity

Locomotive activity was measured during the dark period (12 h), which corresponds to the active period of mice. Activity was counted with an infrared sensor (NS-AS02; Neuroscience Inc., Japan), which was placed 15 cm above the cage top and analyzed using the Digital acquisition system. Activity data were collected at 1-min intervals, and the results were presented as cumulative activity counts over the 12-h dark period. Additional locomotive physical activity was assessed based on wheel-running behavior, using a cage-adjacent wheel (225 × 345 × 210 mm cage with wide 60 × ϕ 140 mm wheel, RWC-15, Melquest Ltd., Japan). The wheel running counts were recorded using a recording and analysis system (CIF-III, Melquest Ltd.). Poly(I:C) (12 mg/kg) was intraperitoneally administered 8 h before the beginning of the dark phase. The activity values are shown as a percentage of those measured one day before administration.

### Evaluation of self-care behavior

Nesting behavior was evaluated 24 h after supplying the nest materials (Nestlets, Animec, Japan). The nesting score, which reflects the degree of nest completion on a 5-point scale, was evaluated as previously reported [36]. Poly(I:C) was administered 8 h before the beginning of the dark phase, after which the nest materials were supplied. Grooming behavior was assessed by recording a video and measuring the grooming time during 5 min after mice were splashed with a solution of 10% (w/v) sucrose on the dorsal surface. The mice were placed in the transparent Plexiglas cylinders (diameter: 12 cm, height: 18 cm) in which they had been acclimated beforehand, as previously reported [37] (representative photograph, Supplementary Fig. S1A). The assessment was performed 6 h after poly(I:C) administration, and each antagonist was administered intraperitoneally 1 h before splashing.

### Measurement of rectal temperature

The rectal temperature of the mice was measured using a small animal thermometer (BWT-100A, Bio Research Center Co., Japan). A small amount of glycerin was coated onto the probe and wiped off after each measurement.

### Measurements of plasma hormone levels

Whole blood was collected from the abdominal vena cava under isoflurane anesthesia, 6 h after poly(I:C) administration. EDTA-2K was used as an anticoagulant during blood collection. The collected blood was immediately centrifuged at 9,700 × g for 3 min to separate plasma, which was then stored at -80 ℃. Plasma hormone concentrations were measured using the enzyme linked immunosorbent assay kits shown in Supplementary Table S2. Samples that were below the detection limit were assigned a value equal to half the detection limit.

### Measurements of hypothalamic genes

Six hours after poly(I:C) administration, hypothalamic tissues were rapidly removed from mice after exsanguination under anesthesia. Total RNA extraction, reverse transcription, and quantitative polymerase chain reaction (PCR) assays were performed using reagents kits shown in Supplementary Table S2 and a QuantStudio™ 7 Flex Real-Time PCR System (Applied Biosystems, USA). mRNA expression was quantified using the ΔΔCt method with gene-specific TaqMan primers and fluorogenic probe sets as shown in Supplementary Table S3.

### In vitro assay

Agonistic activities against human recombinant dopamine D2L and D2S stably expressed in Chinese hamster ovary cells and inhibitory activities against human recombinant MAO-A and B expressed in insect cells were evaluated at Eurofins Panlabs Discovery Services Taiwan, Ltd. (Taipei City, Taiwan, https://apac.eurofinsdiscovery.com/) as previously reported [38–41] (item No. #310,200, #310,300, #140,120, #140,010). NYT (1,000 mg/mL) and its ingredients (100 mM) were pre-dissolved in dimethyl sulfoxide and then diluted to the desired concentrations immediately before the assay.

For D2R agonistic assays, the test compound was preincubated with the membranes and guanosine diphosphate in modified HEPES pH 7.4 buffer for 20 min, and scintillation proximity assay beads were then added for another 60 min at 30 °C. The reaction was initiated by 0.3 nM [^35^S] guanosine 5’-O-[gamma-thio]triphosphate (GTPγS) for an additional incubation period. Quantitation of bound [^35^S] GTPγS was performed by radiometry.

For MAO enzymatic assays, each test compound was preincubated with the enzymes in phosphate buffer pH 7.4 for 15 min at 37 °C. The reaction was initiated by addition of 50 µM kynuramine, incubated for 60 min, and terminated by further addition of 1.2 N NaOH. The amount of 4-hydroxyquinoline formed was measured by spectrofluorimetry.

The results are expressed as the percentage inhibition of the control response to the reference agonist. Concentration–response curve was generated to calculate the half maximal inhibitory concentration (IC_50_) values.

### Statistical analysis

All the results are presented as means ± standard error of the mean. Two-tailed P values were calculated. Multiple comparisons at a single time point were performed using Tukey–Kramer or Steel–Dwass tests. Time-dependent changes were analyzed using two-way repeated-measures analysis of variance (ANOVA) followed by Tukey–Kramer post-hoc tests. Statistical analysis of these data was carried out using GraphPad Prism (GraphPad Software Inc.) or SAS9.4 (SAS Institute Inc.) software. Differences with P < 0.05 were considered statistically significant.

## Results

### Effects of NYT on locomotive activity, wheel-running activity, nesting behavior, and grooming behavior in poly(I:C)-treated mice

We first verified the dose dependency of poly(I:C) based on previous reports [19, 20, 24, 42]. When poly(I:C) was administered intraperitoneally at 3 and 12 mg/kg (frequently reported doses), the locomotive activities of the mice in their home cages decreased in a dose-dependent manner (Supplementary Fig. S2). As a more prominent decrease in locomotive activity was observed at 12 mg/kg, we also assessed wheel-running activity, nesting behavior, and grooming time, which were significantly impaired following 12 mg/kg poly(I:C) administration. Therefore, we investigated the effects of NYT at 12 mg/kg.

**Fig. 2:**
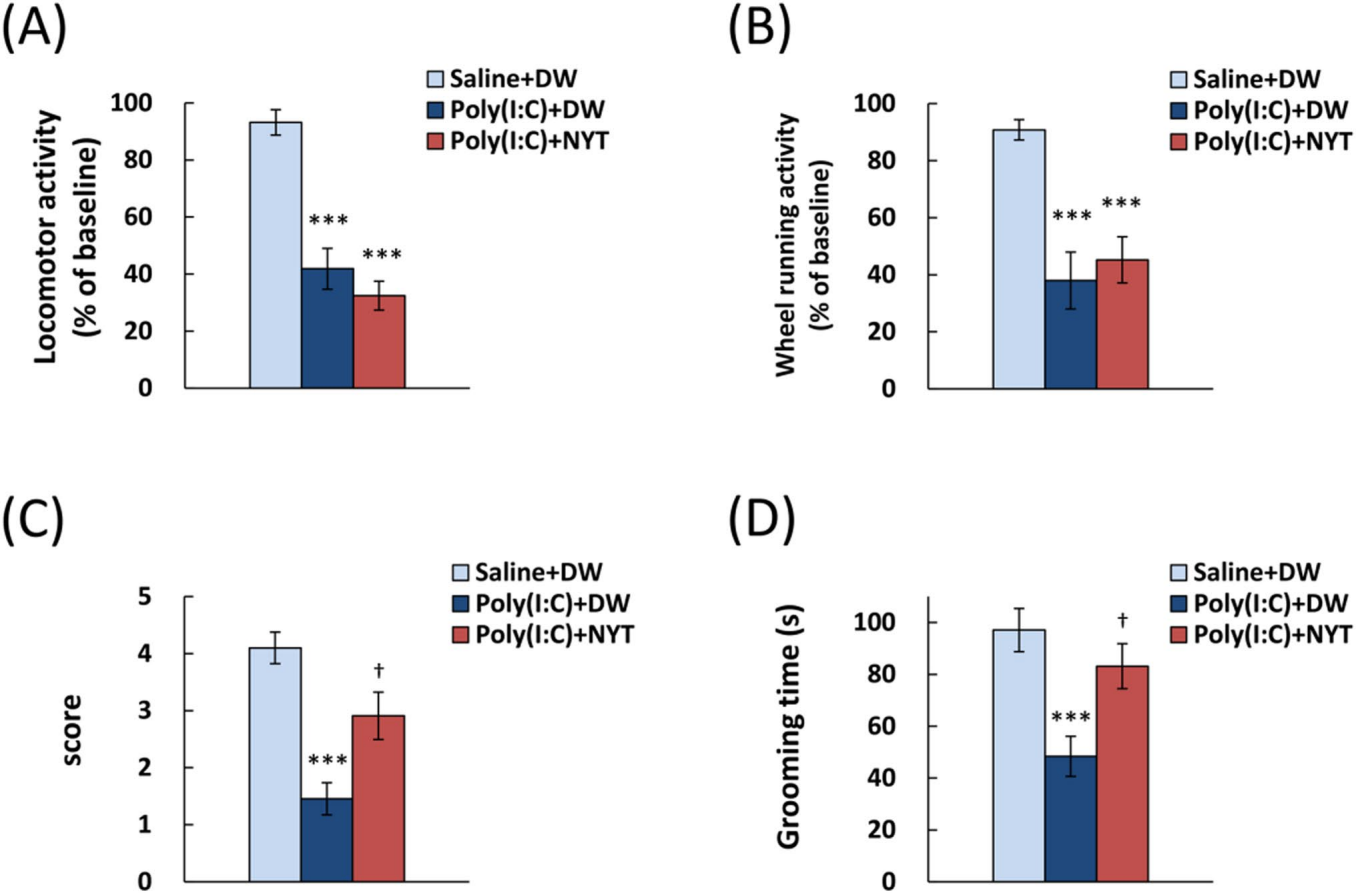
Effect of NYT on impaired activities in poly(I:C)-induced fatigue model mice. Mice were intraperitoneally administered polyinosinic-polycytidylic acid (12 mg/kg) on day 0, and activities were assessed after administration. NYT was administered orally from days -3 to 0 at 1.5 g/kg/day. **A** Effects of NYT on locomotive activity. **B** Effect of NYT on wheel-running activity. **C** Effect of NYT on nest-building behavior. **D** Effect of NYT on the cumulative grooming time 5 min after sucrose splash exposure. Data represent mean ± standard error of the mean, n = 10–11, ***P < 0.001 vs. saline + DW group, †P < 0.05 vs. poly(I:C) + DW group by Tukey–Kramer (**A**, **B**, **D**) or Steel–Dwass (**C**) test. DW: distilled water, Poly(I:C): polyinosinic-polycytidylic acid, NYT: extract powder of Ninjin’yoeito

Although locomotive activity was significantly reduced in poly(I:C)-administered mice, consistent with a previous report [23], NYT showed no ameliorating effect (Fig. 2A). Similarly, NYT did not ameliorate the impaired wheel-running activity (Fig. 2B). In contrast, the decreased nesting behavior score following poly(I:C) administration was significantly restored by NYT (Fig. 2C and S1B). The number of mice with a nesting score ≧ 3 was significantly smaller in the poly(I:C) group than in the vehicle-treated group, and it was significantly larger in the NYT group than in the poly(I:C) group (Supplementary Table S4). NYT significantly reverted the reduction of the grooming time observed following poly(I:C) administration (Fig. 2D).

### Effects of NYT on inflammation in poly(I:C)-treated mice

**Fig. 3:**
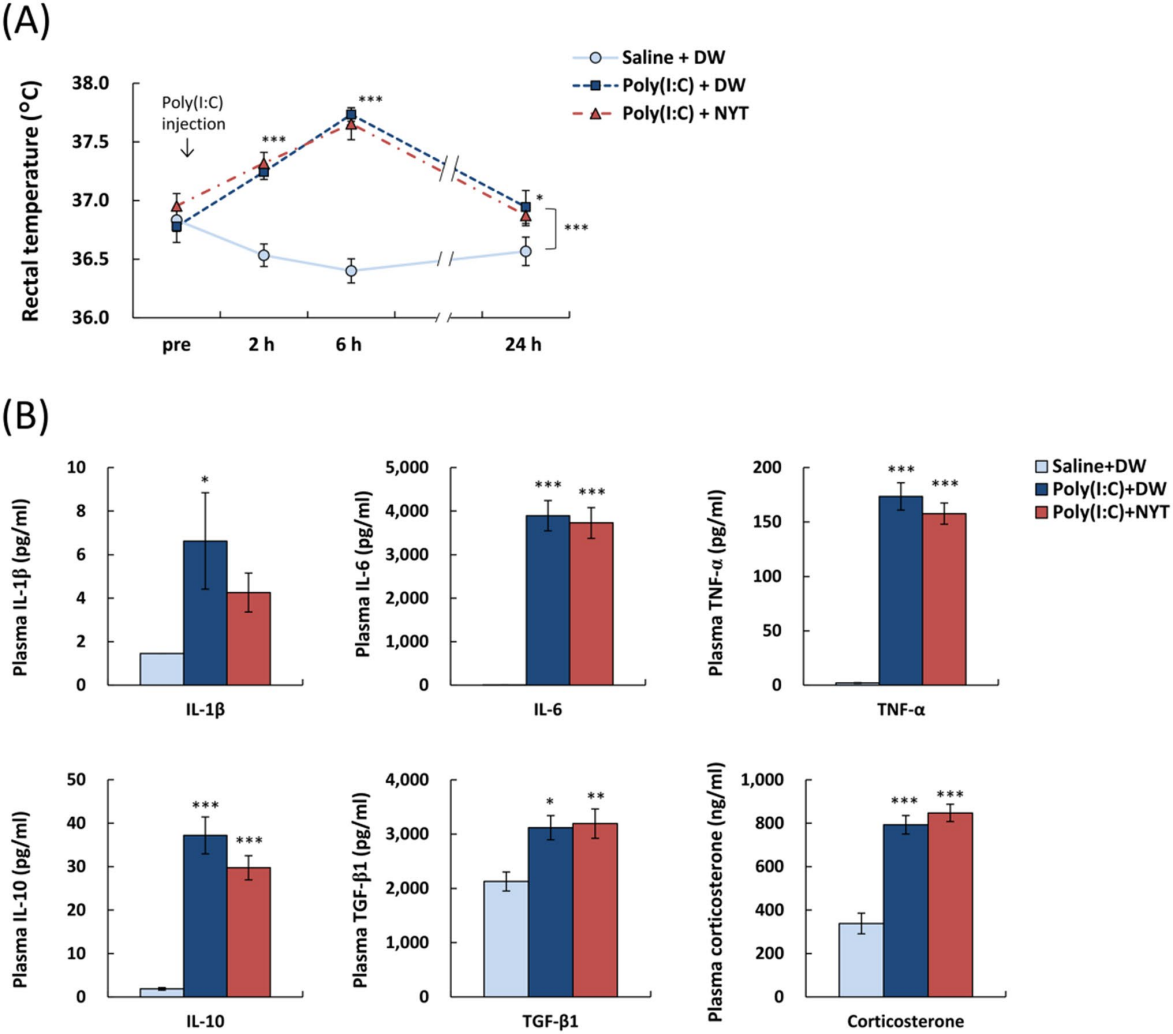
Effects of NYT on inflammation in the poly(I:C)-induced fatigue model mice. **A** Rectal temperature change after poly(I:C)-administration. The effects of time (F (3, 78) = 12.2, P < 0.001), treatment (F (2, 26) = 35.4, P < 0.001), and interaction [time × treatment] (F (6, 78) = 9.12, P < 0.001) were significant by two-way repeated measures analysis of variance. Tukey–Kramer test was performed as a post-hoc test. **B** Plasma hormone levels at 6 h after poly(I:C)-administration. Data represent mean ± standard error of the mean, n = 9–11, *P < 0.05, **P < 0.01, ***P < 0.001 vs. saline + DW group by Tukey–Kramer test. DW: distilled water, Poly(I:C): polyinosinic-polycytidylic acid, NYT: extract powder of Ninjin’yoeito

Next, we verified the effect of NYT on fever and inflammation after poly(I:C) administration. As previously reported [22, 24], the rectal temperature increased after poly(I:C) administration and returned to baseline value 24 h after administration, although it remained significantly higher than that in the vehicle-treated group. However, NYT exhibited no effect on the elevated rectal temperature (Fig. 3A). Likewise, NYT demonstrated no effect on the increased plasma interleukin (IL)-1β, IL-6, tumor necrosis factor (TNF)-α, IL-10, and transforming growth factor (TGF)-β1 levels following poly(I:C) administration (Fig. 3B). Moreover, NYT did not affect the increased cytokine mRNA expression levels in the hypothalamus (decreased only in TGF-β1) following poly(I:C) administration (Table 1). Finally, plasma corticosterone levels, which are involved in stress exposure and inflammatory reactions, were markedly increased by poly(I:C) administration, but remain unaltered by NYT (Fig. 3B).

**Table 1:** Gene expressions in hypothalamuses of poly(I:C)-treated mice

	Il1b	Il6	Tnf	Il10	Tgfb1
Saline + DW	1.00 ± 0.19	1.00 ± 0.05	1.00 ± 0.11	1.00 ± 0.08	1.00 ± 0.03
Poly(I:C) + DW	4.03 ± 0.37^***^	12.5 ± 0.86^***^	10.2 ± 0.50^***^	4.06 ± 0.32^*^	0.58 ± 0.02^***^
Poly(I:C) + NYT	4.03 ± 0.26^***^	13.4 ± 0.75^***^	9.86 ± 0.47^***^	4.99 ± 1.30^**^	0.53 ± 0.01^***^

Gene expression in the hypothalamus of poly(I:C)-treated mice was measured 6 h after administration. Data represent mean ± standard error of mean, n = 9–11; *, **, ***; P < 0.05, 0.01, 0.001 vs. saline + DW group by Tukey–Kramer test. DW: distilled water, Poly(I:C): polyinosinic-polycytidylic acid, NYT: extract powder of Ninjin’yoeito

### Effects of NYT on grooming behavior via dopamine receptor or effect of MAO inhibitor in poly(I:C)-treated mice

**Fig. 4:**
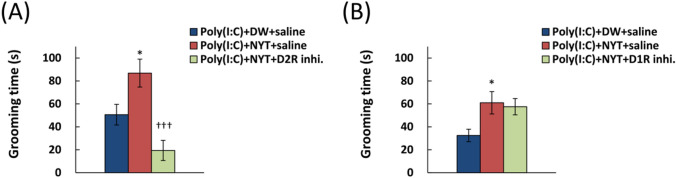
Involvement of dopamine receptor in the restorative effect of NYT in poly(I:C)-induced fatigue model mice. Effects of **A** the dopamine D2 receptor (D2R) inhibitor metoclopramide and **B** the dopamine D1 receptor (D1R) inhibitor SCH-23390 on the effect of NYT on grooming time. Data represent mean ± standard error of the mean. n = 9–11, *P < 0.05 vs. poly(I:C) + DW + saline group, †††P < 0.001 vs. poly(I:C) + NYT + saline group by Tukey–Kramer test. DW: distilled water, Poly(I:C): polyinosinic-polycytidylic acid, NYT: extract powder of Ninjin’yoeito

Since dopamine is involved in the impairment of nesting behavior by water immersion stress exposure [27], we investigated the involvement of D1R and D2R in grooming behavior. The grooming time in the poly(I:C) + NYT-treated group was significantly increased compared to that in the poly(I:C) single-dose group. The NYT effect was completely abolished by co-treatment with the D2R antagonist metoclopramide (Fig. 4A). No significant difference was observed between the poly(I:C) + DW and NYT + antagonist groups. Since the effect of NYT was not eliminated by the D1R antagonist SCH-23390 (Fig. 4B), it was assumed that NYT improved grooming behavior via D2R, but not D1R, in this model.

**Fig. 5:**
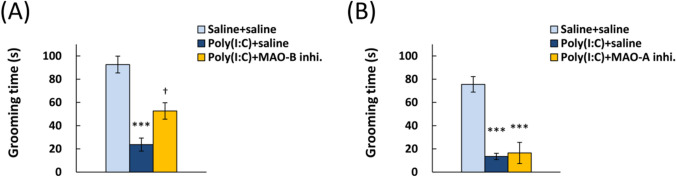
Effect of MAO-B/MAO-A inhibitor on the grooming time reduction by poly(I:C). Effects of **A** monoamine oxidase (MAO)-B inhibitor pargyline and **B** MAO-A inhibitor clorgiline on the grooming time reduction by polyinosinic-polycytidylic acid (poly[I:C]); Data represent mean ± standard error of the mean. n = 9–11, ***P < 0.001 vs. saline + saline group, †P < 0.05 vs. poly(I:C) + saline group by Tukey–Kramer test.

Furthermore, we investigated the effect of MAO inhibitors to examine whether grooming behavior decline was ameliorated by an increase in the dopamine and/or monoamines levels. As a result of MAO-B inhibitor pargyline, the decreased grooming time was significantly prolonged, as was observed with NYT (Fig. 5A). In contrast, the MAO-A inhibitor clorgiline did not affect the grooming time in poly(I:C)-treated mice (Fig. 5B).

### In vitro assay of MAO inhibition by NYT and its ingredients

We assessed the effects of NYT on D2R, dopamine-metabolizing enzymes, and dopamine transporters in vitro. Although NYT 1,000 μg/ml showed no D2R agonistic activity, inhibition was observed against MAO-A by 69%, MAO-B by 95%, catechol O-methyltransferase (COMT) by 54%, and dopamine transporter (DAT) by 100%. Since various ingredients have been reportedly detected in the plasma and brain tissue following NYT administration [43], we examined their inhibitory activities against MAO-A, MAO-B, COMT, and DAT. Formononetin, paeonol, heptamethoxyflavone, and nobiletin were found to show inhibitory activities against MAO-B, with the IC_50_ values of 0.31, 14.7, 65.3, 64.8 μM, respectively (Table 2). Moreover, those ingredients also exhibited inhibitory activities against MAO-A, which is highly specific to serotonin and adrenaline, with the IC_50_ values of 1.72, 18.9, 35.9, and 48.8 μM for formononetin, paeonol, heptamethoxyflavone, and nobiletin (Table 2), respectively. No activity was observed against COMT and DAT (data not shown).

**Table 2:** IC_50_ values of NYT ingredients for MAO-B and MAO-A

Ingredient	Crude drug	IC_50_ for MAO-B	IC_50_ for MAO-A
		(μM)	(μM)
Formononetin Paeonol Heptamethoxyflavone Nobiletin	Astragalus Root Peony Root Citrus unshiu peel Citrus unshiu peel	0.31 14.7 65.3 64.8	1.72 18.9 35.9 48.8

IC_50_: half maximal inhibitory concentration, MAO: monoamine oxidase

Results of positive control: IC_50_ of selegiline for MAO-B; 4.34–6.33 nM, IC_50_ of clorgiline for MAO-A; 0.82–0.97 nM

## Discussion

In this study, we demonstrated for the first time that 1) NYT improved deficits in nesting and grooming behaviors induced by poly(I:C) administration via D2R; 2) Inhibition of MAO-B, rather than MAO-A, similarly improved impaired grooming behavior; and 3) NYT ingredients, such as formononetin and paeonol, may ameliorate behavioral impairments through an increase in dopamine levels.

Previous reports have shown that persistent alterations of locomotive and wheel-running activities in the poly(I:C) model can be restored by intracerebral administration of anti-IL-1β receptor antibody [17, 24] but not by fever suppression using a selective cyclooxygenase-2 inhibitor [24]. Thus, it has been suggested that central inflammation, rather than fever, is strongly involved in the decreased activity by poly(I:C). Notably, in our study, NYT suppressed the deterioration of nesting and grooming behaviors induced by poly(I:C) without affecting the plasma and central levels of inflammatory cytokines, indicating the involvement of other mechanisms.

NYT specifically improved nesting and grooming behaviors without affecting locomotive and wheel-running activities. The former two behaviors are instinctive voluntary survival behaviors in laboratory animals and are considered self-care behaviors [44]. Unlike locomotive and wheel-running activities, which simply indicate the amount of physical activity of the mice, self-care behaviors are clearly motivated [45]. Growing evidence also suggests that dopamine is important for grooming and nesting behaviors [46, 47]. A previous study reported an association between grooming behavior and D1R and/or D2R [48–51]. A cooperative interaction between the D1R and D2R systems has been proposed to play a role in the regulation of grooming behavior [52], although the details remain to be elucidated. Serafim et al. demonstrated that the administration of a D2R antagonist significantly decreased the physiological grooming time in non-pregnant female mice [53], and the predominance of D2R in regulating grooming behavior has also been reported. In this study, co-treatment with a D2R antagonist, rather than D1R, blocked the ameliorative effect of NYT on grooming behavior. The results suggest that NYT restores physiological grooming behavior deficits through D2R activation. In addition, nesting behavior, also known as goal-directed behavior, is mediated by dopaminergic neurons, which project from the substantia nigra into the striatum [46]. Dopamine-deficient mice also show impaired nesting behavior [47]. We have previously shown that impaired nesting behavior was improved by NYT via D2R in mice exposed to water immersion stress [27]. Collectively, these findings suggest that D2R activation may represent one of the key mechanisms by which NYT improves motivational behaviors related to life maintenance, such as nest building.

NYT may increase D2R dopamine signaling by elevating the dopamine levels in the synaptic cleft. NYT and its ingredients (onjisaponin B, nobiletin, and schizandrin) reportedly increase the levels of dopamine and decrease those of its metabolites, namely, 3,4-dihydroxyphenylacetic acid (DOPAC), and 3-methoxytyramine, in neuron PC12 dopamine-producing cells [54]. Additionally, NYT ingredients, such as calycosin, hesperetin, onjixanthone II, tenuifoliside, onjisaponin, 3,6'-disinapoyl sucrose, and catalpol, showed inhibitory activity against MAO-B, COMT, and DAT in vitro [27, 55, 56]. In this study, we showed that formononetin, paeonol, heptamethoxyflavone, and nobiletin—ingredients known to reach the brain following NYT administration—exhibited inhibitory activity against MAO-B in vitro. The inhibitory effect of formononetin was consistent with the results of a previous study [57]. However, compared with selegiline, the positive control drug, the IC₅₀ values of the individual ingredients identified in this study were markedly higher. Therefore, the concentrations of these ingredients in the brain following NYT administration [43] are unlikely to reach pharmacologically effective levels. It is suggested that the effect of a single ingredient may be limited. Thus, multiple ingredients may act additively or synergistically to exert the observed effect, and contributions from ingredients that were unknown and/or unmeasured in this study cannot be excluded. In addition, a previous study reported that both MAO-B and DAT inhibitors ameliorated the impairment in nest-building behavior in stress-loaded mice [58]. Inhibition of COMT and DAT—both of which may increase dopamine levels, similar to MAO-B inhibition—may exert additional effects, since NYT ingredients such as onjisaponin B and onjixanthone II possess inhibitory activities against them.

Poly(I:C) reportedly reduces the serotonin levels in the prefrontal cortex [20], and serotonin is also known to modulate nesting and grooming behaviors [59–62]. However, inhibition of MAO-A, which primarily metabolizes serotonin, did not restore poly(I:C)-induced grooming deficits. Thus, the role of dopamine may be more attributable to the improving effect of NYT rather than serotonin.

Furthermore, the dopamine levels in the striatum showed no substantial changes among the groups at 6 h after poly(I:C) administration (data not shown). While the levels of the dopamine metabolites DOPAC and homovanillic acid (HVA) were significantly increased in the NYT-treated group compared with the untreated group (ratio of peak area [target/internal standard] for DOPAC: Saline + DW; 0.41 ± 0.03, Poly(I:C) + DW; 0.60 ± 0.06, Poly(I:C) + NYT; 0.97 ± 0.10, P < 0.01 vs. Saline + DW and Poly(I:C) + DW, HVA: Saline + DW; 0.28 ± 0.03, Poly(I:C) + DW; 0.39 ± 0.05, Poly(I:C) + NYT; 0.78 ± 0.11, P < 0.01 vs. Saline + DW and Poly(I:C) + DW by liquid chromatography-mass spectrometry). These results indicate that dopamine levels increased, even if only temporarily. They may also suggest an enhancement of dopamine turnover. One possible interpretation is that NYT exerts relatively weak inhibitory effects on MAO, as suggested by the brain distribution of its ingredients and their IC₅₀ values. Under these conditions, NYT may partially attenuate dopamine metabolism, yielding only a transient increase in extracellular dopamine. The observed increase in metabolites may therefore reflect the remaining MAO activity. Given that dopaminergic neurotransmission fluctuates rapidly in a time-dependent manner, we cannot exclude the possibility that the sampling time point used in this study was not optimal.

This study has certain limitations. We were unable to obtain direct evidence for dopamine alterations associated with poly(I:C)-induced impairment of self-care behavior, or for NYT-induced increases in dopamine levels. Further investigation is required to elucidate the mechanisms through which NYT activates the D2R pathway. Monitoring temporal changes in extracellular dopamine levels in the brain using microdialysis, followed by time-matched analyses of D2R pathway activation (e.g., the measurement of downstream targets such as the phosphorylation of AKT/GSK‑3β and cAMP), may help clarify the mechanisms of action of poly(I:C) and NYT. In addition, further investigations are required to examine the potential contributions of COMT and DAT within this model in future research.

## Conclusion

Our study demonstrated that NYT may ameliorate certain fatigue-like behaviors—specifically, impaired nesting and grooming—by stimulating D2R through inhibition of dopamine metabolism in a poly(I:C)-induced inflammatory fatigue mouse model.

## Supplementary Information

Below is the link to the electronic supplementary material.

Supplementary file1 (PDF 390 KB)

## Data Availability

All relevant data in the study are included in the article/supplementary material. Further inquiries can be directed to the corresponding author.
