# Supplementary material for: Ninjin’yoeito ameliorates deficits in self-care behaviors in a polyinosinic-polycytidylic acid-induced fatigue model via dopamine D2 receptor activation
Source: J Nat Med. 2026 Jun 2;80(4):1026–36. doi: 10.1007/s11418-026-02035-4 (PMC13350208; doi:10.1007/s11418-026-02035-4)
Supplement: Supplementary file 1 — Supplementary file1 (PDF 390 KB) [file 11418_2026_2035_MOESM1_ESM.pdf]

Supplementary Information

Supplementary Figures

(A)

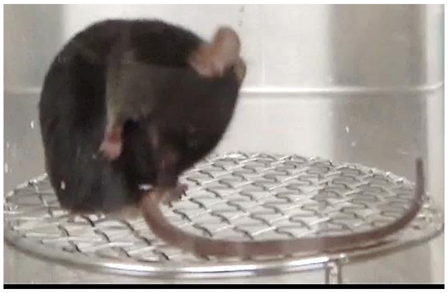

(B)

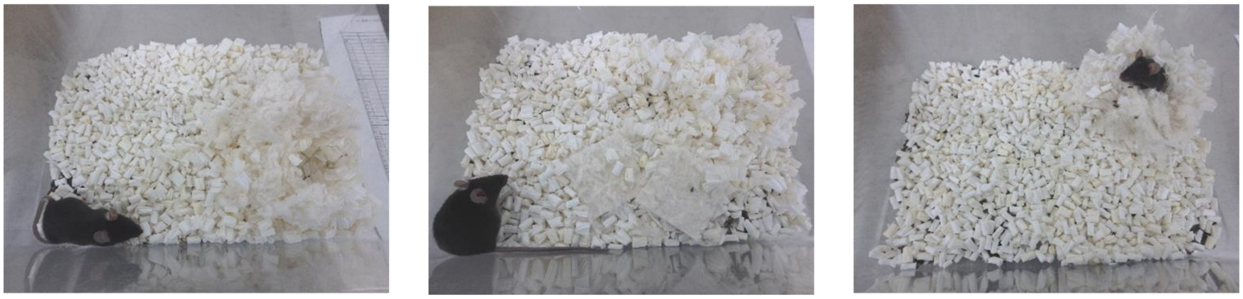

Saline + DW　　Poly(I:C) + DW　　Poly(I:C) + NYT

Supplementary Figure S1. Representative photograph of (A) grooming behavior and (B) nest-building behavior. DW: distilled water, Poly(I:C): polyinosinic-polycytidylic acid, NYT: Ninjin'yoeito extract powder

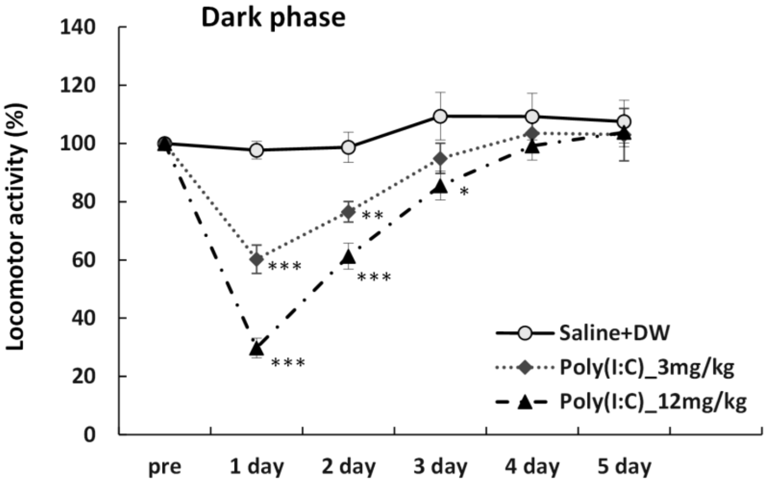

Supplementary Figure S2. Locomotive activity after poly(I:C) administration. The activity values are shown as a percentage of those measured one day before administration. Data represent mean ± standard error of mean, n = 10–11, * ** ***; $P < 0.05, 0.01, 0.001$ vs. saline + DW group by Dunnett test. DW: distilled water, Poly(I:C): polyinosinic-polycytidylic acid

Supplementary Table S1. The composition ratio of crude drugs in Ninjin'yoeito

Crude drugs	Botanical source	composition ratio
JP Rehmannia root	*Rehmannia glutinosa* Liboschitz var. *purpurea* Makino or *Rehmannia glutinosa* Liboschitz (*Scrophulariaceae*)	4.0
JP Japanese angelica root	*Angelica acutiloba* Kitagawa, or *Angelica acutiloba* Kitagawa var. *sugiyamae* Hikino (*Umbelliferae*)	4.0
JP Atractylodes lancea rhizome	*Atractylodes lancea* De Candolle, or *Atractylodes chinensis* Koidzumi or their interspecific hybrids (*Compositae*)	4.0
JP Poria sclerotium	*Wolfiporia cocos* Ryvarden et Gilbertson (*Poria cocos* Wolf) (*Polyporaceae*)	4.0
JP Ginseng	*Panax ginseng* C.A. Meyer (Araliaceae), radix	3.0
JP Cinnamon bark	*Cinnamomum cassia* J. Presl (Lauraceae)	2.5
JP Polygala root	*Polygala tenuifolia* Willdenow (Polygalaceae)	2.0
JP Peony root	Paeonia lactiflora Pallas (Paeoniaceae)	2.0
JP Citrus Unshiu peel	*Citrus unshiu* Marcowicz or *Citrus reticulata* Blanco (*Rutaceae*)	2.0
JP Astragalus root	*Astragalus membranaceus* Bunge or *Astragalus mongholicus* Bunge (Leguminosae)	1.5
JP Glycyrrhiza	*Glycyrrhiza uralensis* Fischer or *Glycyrrhiza glabra* Linné (*Leguminosae*), radix	1.0
JP Schisandra fruit	*Schisandra chinensis* Baillon (*Schisandraceae*)	1.0

JP: Japanese Pharmacopoeia

Supplementary Table S2. Reagents and kits.

Product name	Manufacture	Catalog No.
Mouse TGF-beta 1 Quantikine ELISA Kit	R&D Systems, Inc., USA	MB100B
AssayMax Corticosterone ELISA Kit	ASSAYPRO, USA	EC3001-1
Mouse Cytokine/Chemokine Magnetic Bead Panel kit	Merck KGaA, Germany	MCYTOMAG-70K
RNeasy Universal Tissue kits	Qiagen, Germany	73404
TaqMan Reverse Transcription Reagents kits	Applied Biosystems, USA	4304134
TaqMan Fast Advanced Master Mix	Applied Biosystems	4444965

Supplementary Table S3. TaqMan primers and fluorogenic probe (Applied Biosystems)

Target	Catalog No.
Rps29	Mm02342448_gH
Il1b	Mm00434228_m1
Il6	Mm00446190_m1
Tnf	Mm00443258_m1
Il10	Mm01288386_m1
Tgfb1	Mm01178820_m1

Supplementary Table S4. Number of mice with a nesting score ≧ 3.

Score	Saline + DW	Poly(I:C) + DW	Poly(I:C) + NYT
≧ 3	10	1 ***	7 ††
< 3	0	10	4
Total number	10	11	11

Effect of NYT on decreased nesting score in poly(I:C)-treated mice. n = 10–11, ***; $P < 0.001$ vs. saline + DW group, ††; $P < 0.01$ vs. poly(I:C) + DW group by chi-squared test. DW: distilled water, Poly(I:C): polyinosinic-polycytidylic acid, NYT: Ninjin’yoeito extract powder
